# Risk Assessment of Gastric Cancer Caused by *Helicobacter pylori* Using CagA Sequence Markers

**DOI:** 10.1371/journal.pone.0036844

**Published:** 2012-05-15

**Authors:** Chao Zhang, Shunfu Xu, Dong Xu

**Affiliations:** 1 Department of Computer Science, University of Missouri, Columbia, Missouri, United States of America; 2 C.S. Bond Life Science Center, University of Missouri, Columbia, Missouri, United States of America; 3 Department of Gastroenterology, The First Affiliated Hospital of Nanjing Medical University, Nanjing, Jiangsu, China; University of Hyderabad, India

## Abstract

**Background:**

As a marker of *Helicobacter pylori*, Cytotoxin-associated gene A (cagA) has been revealed to be the major virulence factor causing gastroduodenal diseases. However, the molecular mechanisms that underlie the development of different gastroduodenal diseases caused by cagA-positive *H. pylori* infection remain unknown. Current studies are limited to the evaluation of the correlation between diseases and the number of Glu-Pro-Ile-Tyr-Ala (EPIYA) motifs in the CagA strain. To further understand the relationship between CagA sequence and its virulence to gastric cancer, we proposed a systematic entropy-based approach to identify the cancer-related residues in the intervening regions of CagA and employed a supervised machine learning method for cancer and non-cancer cases classification.

**Methodology:**

An entropy-based calculation was used to detect key residues of CagA intervening sequences as the gastric cancer biomarker. For each residue, both combinatorial entropy and background entropy were calculated, and the entropy difference was used as the criterion for feature residue selection. The feature values were then fed into Support Vector Machines (SVM) with the Radial Basis Function (RBF) kernel, and two parameters were tuned to obtain the optimal F value by using grid search. Two other popular sequence classification methods, the BLAST and HMMER, were also applied to the same data for comparison.

**Conclusion:**

Our method achieved 76% and 71% classification accuracy for Western and East Asian subtypes, respectively, which performed significantly better than BLAST and HMMER. This research indicates that small variations of amino acids in those important residues might lead to the virulence variance of CagA strains resulting in different gastroduodenal diseases. This study provides not only a useful tool to predict the correlation between the novel CagA strain and diseases, but also a general new framework for detecting biological sequence biomarkers in population studies.

## Introduction

*Helicobacter pylori (H. pylori)* is a Gram-negative helix-shaped bacterium inhabiting the human stomach and infecting more than half of the world’s population [1], [2], [3]. Recent studies have shown that it is associated with gastroduodenal diseases, including duodenal ulcers [4], gastric ulcers [5] and chronic gastritis. More importantly, it is a significant risk factor for developing gastric cancer [6], [7], [8]. It has been classified as a Class 1 human carcinogen by the World Health Organization since 1994 [1].

As a marker of *H. pylori*, the Cytotoxin-associated gene A (cagA) has been revealed by further analysis to be the major virulence factor. *H. pylori* strains carrying the cagA gene increase the risk factor of gastroduodenal diseases by three folds over cagA-negative strains [6], [9], [10]. CagA, which is encoded by the cagA gene, is a 125–140 kDa protein. It contains 1142–1320 amino acids and has a variable region at the C-terminal region in which various short sequences (such as EPIYA motif) repeat 1–7 times. After *H. pylori* colonizing on the surface of the gastric epithelium, CagA can be translocated into the gastric epithelial cell through a type IV secretion system. Once injected into the host cell, CagA localizes to the plasma membrane and can be phosphorylated by Src-family tyrosine kinases on the specific tyrosine residues of a five-amino-acid (EPIYA) motif [11], [12], [13], [14]. Tyrosine-phosphorylated CagA then binds specifically to SHP-2 tyrosine phosphatase 11,15 to activate a phosphorylase, which causes the cascade effect that interferes with the signal transduction pathway of the host cell, leading to a restructuring of the host cell cytoskeleton and formation of hummingbird phenotype [11], [16]. At the same time through activating mitogen-activated protein kinase (MAPK), extracellular signal-regulated kinase (ERK) [17] and focal adhesion kinase (FAK), CagA also can cause cell dissociation and infiltrative tumor growth [18], [19], [20], [21]. Such a process makes CagA a most important virulence factor in *H. pylori* [22].

Within the variable region of CagA, there are some different intervening sequences between those EPIYA motifs. One copy of EPIYA plus intervening sequence is identified as an EPIYA segment. Four unique types of EPIYA segments have been found in CagA, defined as EPIYA-A, -B, -C and -D [11]. The CagA isolated from East Asian countries, designated as East Asian CagA, contains EPIYA-A, EPIYA-B and EPIYA-D motifs. The CagA from Western countries, EPIYA-D, is replaced by EPIYA-C. Stronger phosphorylation motif binding activity of the EPIYA-D motif leads to greater morphological changes than what the EPIYA-C motif can cause in infected cells [11]. It is this EPIYA-D motif’s increased binding activity and resultant morphological changes that identifies it as a potential factor to explain the higher incidence of gastric cancer in East Asian countries [23], [24].

Previous studies revealed a variation in the number of EPIYA motif repeats for both East Asian and Western CagA, which can affect biological activities. Yamaoka et al. [25] found that in Columbia and USA, the ability of cagA-positive *H. pylori* to cause gastric mucosal atrophy and intestinal metaplasia might be related to the number of EPIYA motifs in the CagA strain. Argent et al. [16] came to the same conclusion later. However, contrary opinions were published by Lai et al. [26] based on findings of no relationship between the number of EPIYA motifs in the CagA strain and clinical disease within 58 isolates from Taiwan. Considering the size and geographic limitation of these studies, the validity of this conclusion is questionable. Aside from the number of the EPIYA motif repeats, the sequence difference of strains in variable regions also could cause a significant difference of virulence, which might relate to the different pathogenic abilities of *H. pylori* [27].

Because of the complex and variant sequences in CagA, the relationships between the polymorphism of CagA and clinical diseases become a very interesting research problem. However, the molecular mechanisms that underlie different gastroduodenal diseases caused by cagA-positive *H. pylori* infection remain unknown. Until now most studies are still limited to the discovery or evaluation of the correlation between the number of CagA EPIYA motifs and diseases [28].

In this paper, we propose a systematic method to analyze not only the number of EPIYA motifs in CagA sequences but also the specific sequence patterns of intervening regions. First, we introduce entropy calculation to detect the residues within the variable region of CagA as the gastric cancer biomarkers. Then we employ a supervised learning procedure to classify cancer and non-cancer by using the information of detected residues in CagA as the features. We choose support vector machines (SVM) as a binary classifier and compare our method with others. Our approach not only proves our hypothesis that the sequence of variable region of CagA contains information to distinguish different diseases, but also provides a useful tool to predict the correlation between the novel CagA strains and diseases and to detect the biomarker as well.

**Figure 1:**
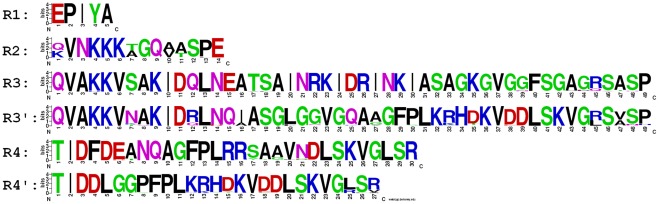
Profiles of the CagA repeat regions.

**Figure 2:**
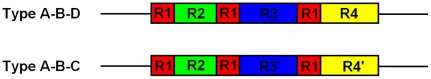
Structure examples: A-B-D and A-B-C types of CagA sequences (not on a proportional scale to sequence length).

## Methods

### Data Preprocessing

Based on the previous description in Ref. [15], we named the EPIYA motif and the following intervening regions R1, R2, R3, R3′, R4 and R4′ (Figure 1). Figure 2 shows the position relation between the EPIYA motif (R1) and other intervening regions by using the CagA types A-B-D (East Asian subtype) and A-B-C (Western subtype) as examples. R2 is relatively conserved across both subtypes, but there are significant differences between the intervening regions R3 and R3′, as well as between R4 and R4′. The East Asian subtype and the Western subtype were treated as two independent groups. Their data was then processed and the results were analyzed within each group individually.

All intervening regions were extracted from the CagA sequences and put into the corresponding subtype groups, and then the multiple sequence alignments were applied for each group individually by using Clustal X version 2.0.3 [29]. The sequences profiles (Figure 1) was built by using the Weblogo 3 [30].

**Figure 3:**
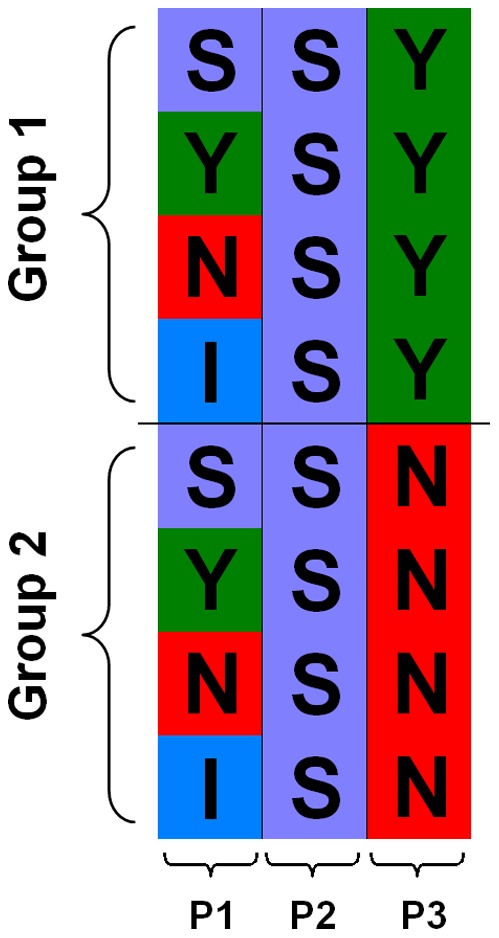
An example to present different cases for the entropy calculation.

### Residue Detection

Since CagA is related to almost all gastroduodenal diseases and simple analysis of EPIYA motif repeats does not yield any statistically significant differences among those diseases, the information indicating a specific disease might be hidden in the intervening regions. This research assumes that there is a set of residues or residue combinations that could be useful as a marker of a specific disease. This study focuses on the gastric cancer and uses the cancer/non-cancer groups as the example.

**Figure 4:**
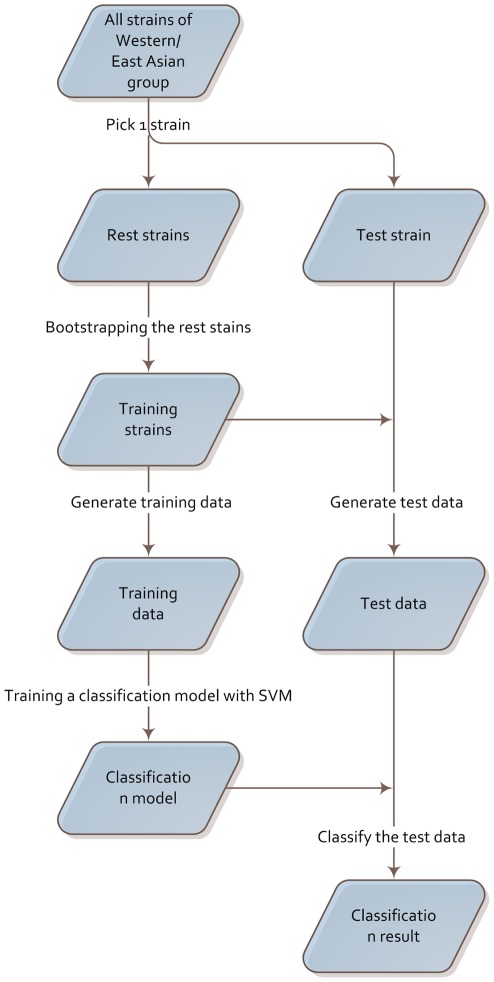
Workflow of classification/prediction procedure for one specific CagA sequence.

Based on the aligned sequences for each intervening region, specific residues were identified by comparing the difference of combinatorial entropy [31] between the cancer and non-cancer groups. This procedure includes the following steps:

First of all, we divide the given multiple alignments for all intervening regions into two groups: gastric cancer group and non-cancer group. For each column of multiple alignments, we compute the background entropy (Eq. 1) and the combinatorial entropy (Eq. 2), described as follows:
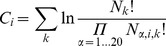
(1)where 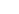 represents the number of sequences in group *k*. 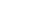 indicates the number of residues of type 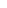 in the column *i* of group *k*. 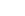 is the number of residues of type 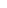 in the column *i*. 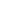 represents the total number of sequences in alignment.
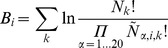
(2)where 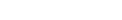.

Then the entropy difference between the combinatorial entropy and the background entropy is calculated:
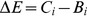
(3)


Figure 3 illustrates the entropy concept using three extreme cases. In case P1, the amino acids are ‘randomly and uniformly distributed’ over all groups and there is no significantly conserved pattern for this position. Case P2 represents a ‘globally conserved’ pattern and all the amino acids are the same across both groups. In case P3, some specific amino acids are only conserved in particular groups, and different groups have different amino acids. We call this case ‘locally conserved’.

According to the calculation results of the entropy difference for the above three cases, the combinatorial entropy is 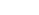 for both ‘globally conserved’ and ‘locally conserved’ cases. For ‘randomly and uniformly distributed’ case, 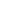 gets the maximum value. We can distinguish the ‘conserved’ and ‘randomly and uniformly distributed’ cases based on the combinatorial entropy, but it does not help pick ‘locally conserved’ case from all ‘conserved’ cases. When we consider the background entropy at the same time, 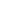 gets the maximum value, 0 and medium value for the ‘randomly and uniformly distributed’ case, ‘globally conserved’ case, ‘locally conserved’ case, respectively. Finally, the differences for the above three cases are: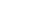, 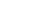, and 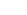 gets the minimum value. Hence, the entropy difference is a proper measurement for detecting a ‘locally conserved’ sequence pattern.

### Feature-entropy Calculation

Based on the above calculation, it can be determined that correct grouping can minimize the entropy difference for those residues belonging to the ‘locally conserved’ case. To perform a test, one sequence is selected while the rest of the sequences are divided into a gastric cancer group and a non-cancer group. For all selected residues, the selected sequence is placed into the gastric cancer group to calculate the entropy difference 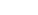, and then it is placed into non-cancer group to get the corresponding entropy difference 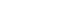. Finally, 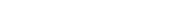 is obtained for all selected residues that are used as the feature entropy.

### Classification of CagA Sequences

#### Dataset

We searched the National Center for Biotechnology Information (NCBI), the Swiss-prot/Tremble and DDBJ protein database and obtained 535 strains of *H. pylori* CagA protein. Among them, there are 287 East Asian subtype strains and 248 Western subtype strains. In the East Asian subtype group, 47 out of 287 strains are from gastric cancer patients and the rest are from other diseases. In the Western subtype group, there are 37 strains from the gastric cancer patients, and the remainders are from other diseases or the normal controls, including 24 strains from volunteers whose health (disease) status was unknown.

#### Workflow

Figure 4 shows the workflow of the classification/prediction procedure:

- Select one strain as the test strain.
- Apply a bootstrap procedure to the rest of the strains to get the training strains.
- Calculate the feature entropy for the test strain based on training strains and save it as the test data.
- Calculate the feature entropy for each strain in the training strain set based on training strains and save them as the training data.
- Generate classification model by using the training data.
- Classify the test data according to the classification model.
- Repeat this procedure five times, and then calculate the average as the final result.

#### Bootstrapping

A major issue in building a classification model in this case is the big difference of the sample sizes between cancer and non-cancer groups, which could cause bias in the classification results. A bootstrapping procedure was applied to address this issue. In each subtype group, for each training/test data sets, all non-cancer samples were included, and then strains were continuously drawn from the cancer group on a random basis until reaching the same size of the non-cancer group. In this case, all the available data were used although cancer samples were utilized multiple times given their smaller size compared to the non-cancer group. This procedure was applied five times to generate five independent training sets for each test sequence. The classification/prediction result is the average of those five independent results.

#### Cross-validation

Because the data size is small, a leave-one-out (LOO) cross-validation procedure was performed. This is not only an assessment of the classifier performance on training/test data, but also an estimate of prediction power for novel cases.

#### SVM

We chose SVM as binary classifier and used the feature-entropy vectors to train and test the classifier. In the case of two-class soft margin classification, the decision function is a weighted linear combination defined as follows:
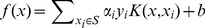
(4)where 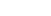 represents a user-defined kernel function that measures the similarities between the input feature vector 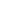 and the feature vectors 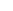 in the training dataset 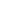. 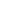 is the weight assigned to the training feature vector 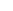 and 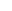 indicates whether a CagA strain has been labeled with the positive class (+1) or negative class (−1). The primal optimization problem takes the form:minimize
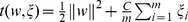
(5)subject to
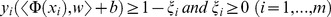
(6)where 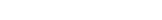. m is the total number of strains. 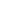 is a slack variable which measures the degree of misclassification of the datum. 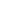 is a cost parameter which allows for trading off training error against model complexity. w is the normal vector and b is the offset.

After comparing the results of polynomial, tanh and Gaussian radial basis kernels, the result obtained with the RBF kernel worked the best, where the Gaussian radial basis kernels (RBF: 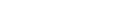) are for general-purpose learning when there is no prior knowledge about the data. The SVM^Light^ package (http://svmlight.joachims.org/) [32] was employed to build our application. The parameters 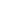 and 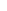 were tuned to get the best model for the training data as shown in the following. All other SVM parameters were set to their default values.

**Figure 5:**
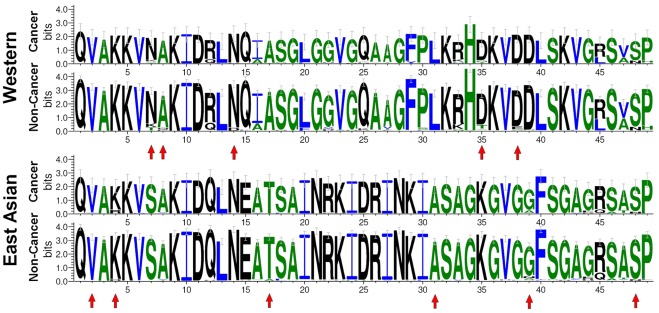
Comparison of sequence profiles between cancer and non-cancer groups and selected features (residues marked with arrows) based on entropy calculation in the R3'/R3 region.

#### Performance evaluation

In order to evaluate the performance of classifier, a variety of performance measures are applied: accuracy, sensitivity and specificity. A true positive (TP) is a cancer-related sequence classified as such, while a false positive (FP) is a non-cancer related sequence classified as cancer-related, a false negative (FN) is a cancer related sequence classified as non-cancer related and a true negative (TN) is a non-cancer related sequence classified as non-cancer related. The accuracy, sensitivity (Sn), specificity (Sp) and Matthews correlation coefficient (MCC) of classification is defined as follows:
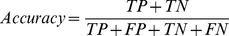
(7)

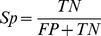
(8)

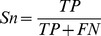
(9)

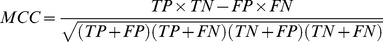
(10)Since there are only two parameters 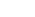 for the RBF kernel and they are independent, we applied a grid-search to determine the optimal parameters of classifier. We used a harmonic means of sensitivity and specificity as the objective function to optimize the performance of the model for the training set, which is defined as follows:


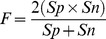
(11)

## Results

### Residue Detection and Feature Calculation

Table 1 lists all detected key residues by calculating the entropy difference in each intervening region for both Western and East Asian subtypes. Although there are some geographic variations of CagA sequences between the Western and East Asian subtypes, some common residues could still be found to distinguish the cancer and non-cancer groups. It suggests that those residues might be very important in determining the virulence of CagA and the relation between CagA and some specific diseases.

**Table 1:** Detected residues by calculating entropy difference for each intervening region between Western and East Asian subtypes.

Intervening regions	Western subtype	East Asian subtype
R2	1, 8, 10, 12	1, 8, 10, 12
R3'/R3	7, 8, 14, 35, 38	2, 4, 17, 31, 39, 48
R4'/R4	9, 14	16

The residue positions are shown in Figure 5. A previous study [27] reveals that the different EPIYA segments can bind to the different kinases, e.g., EPIYA-R2 and EPIYA-R3/R3′ bind to the C-terminal Src kinase (Csk) while EPIYA-R4 and EPIYA-R4′ bind to the SHP-2 kinase to cause the hummingbird phenotype. The CagA-Csk interaction down-regulates CagA-SHP-2 signaling that perturbs cellular functions to control the virulence of CagA. It is found that most detected residues belong to R2 and R3/R3′ regions and few residues in R4/R4' regions have been detected. This may be because R4/R4' has more conserved sequence than R2, and R4/R4′ is shorter than R3/R3′. We suggest that the different residue patterns in R2 or R3/R3′ regions might change the ability of down-regulating CagA-SHP-2 signaling, therefore changing the virulence of CagA.

Ren et al. found that CagA multimerizes in mammalian cells [33]. This multimerization is independent to the tyrosine phosphorylation, but it is related to the “FPLxRxxxVxDLSKVG” motif which is named CM motif in the R3′ intervening region. Since the multimerization is a prerequisite for the CagA-SHP-2 signaling complex and subsequent deregulation of SHP-2, the CM motif plays an important role in cagA-positive *H. pylori*-mediated gastric pathogenesis. With multiple CM motifs *H. pylori* strains are much likely associated with severe gastroduodenal diseases [33], [34], but this observation cannot explain why different gastroduodenal diseases can be developed with the exact same number of CM motifs. Our study detected two residues in the CM motif of R3′ intervening region, which might lead to the change of multimerization, thus changing the virulence of CagA. This is in consistent with a previous discovery [35] that the sequence difference between the East Asian CM and the Western CM determines the binding affinity between CagA and SHP-2.

While the key residues detected can reveal some difference between cancer and non-cancer groups, no single residue can be a marker for cancer as shown in Figure 5. This research predicts that one special combination of all or partial detected residues could have a high correlation with one particular disease. To verify, several linear statistical models, e.g. linear regression and logistic regression, were applied to the detected features to evaluate the importance of each residue and the correlation between selected residues and cancer. However, none of above models were able to produce a statistically significant result. Since the features cannot be fitted by simple linear models for predicting cancer, applying a machine learning method to analyze and classify these data becomes necessary.

### Parameter Training for Classification

**Figure 6:**
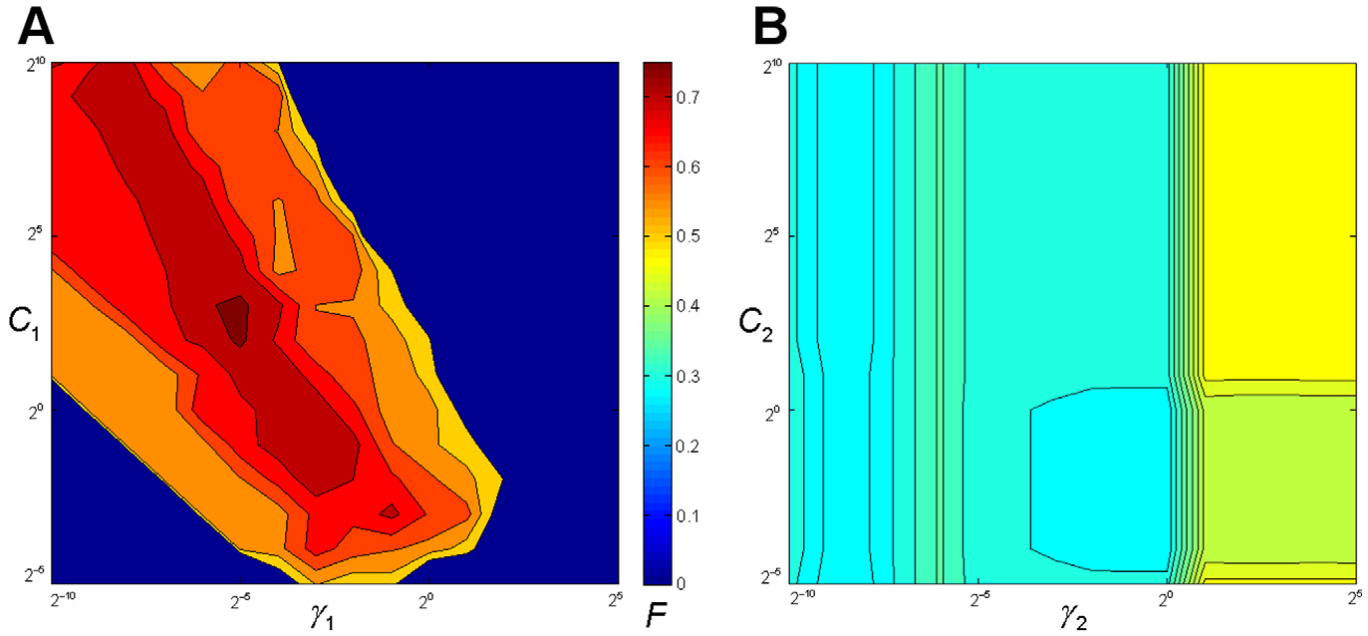
Grid-search for determining the optimal parameters 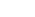 of classifier, with color indicating the F value. (A) The contour plot of F value resulting from a loose grid-search on a hyper parameter range for the Western subtype group. (B) The contour plot of F value resulting from a loose grid-search on a hyper parameter range for a randomly shuffled Western subtype group with the highest F value.

Using the Western subtype group as the example, a loose grid-search was first performed on 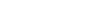 and 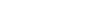 (Figure 6A) and found that the best 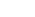 is around 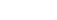 to get the highest F value with the LOO cross-validation rate 76%. Then a finer grid search was conducted on the neighborhood 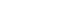 and a better F value was obtained with 79.7% LOO cross-validation at 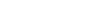. The same procedure was utilized for the East Asian subtype group and the best LOO cross-validation rate 72.6% was reached at 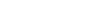.

Since there are no previous studies or computational methods on the same topic, evaluating the performance of this research’s new method is difficult. To assess the information content of the sequences in terms of their discerning power to predict cancer, a random shuffling procedure was employed to build the control group. First, all sequences from the Western subtype were placed together to build a sequence pool. Second, we randomly picked the same number of sequences as cancer group from the sequence pool and treated the rest of the sequences as the non-cancer group. Then, the whole training procedure was applied to newly shuffled data to find the best 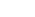. The above steps were repeated five times to generate five independent shuffled data sets. The one with the highest *F* value, which equals 46.6% was selected and its contour plot is shown in Figure 6B. This randomly shuffling evaluation was also applied to the East Asian subtype data and the best *F* value was at 54.3%. Comparing the two plots shows the significant difference of *F* values between the data with correct grouping of cancer and non-cancer cases in training and the best randomly shuffled data. The result suggests that the intervening regions are informative to distinguish between the cancer and non-cancer groups and our method can use the information effectively.

### Classification Performance

There are mainly three categories of sequence classification methods: feature based, sequence distance based and model based. The method that we described in this paper belongs to the feature-based category. We selected two of the most popular sequence classification tools as the representative methods of other two categories for comparison. BLAST [36] was chosen for the sequence distance based category, since it is the most widely used sequence comparison tool. For the model-based category, the hidden Markov model is the typical method for sequence analysis and its widely used tool, HMMER [37], was selected. For the classification procedure of both BLAST and HMMER, we used the default parameters of the tools, applied the same LOO cross-validation as our method, and used the same evaluation formulas listed in the Method section.

Table 2 lists the classification results for all three methods. The SVM method performs significantly better than the other two approaches. BLAST achieved close accuracy to the Entropy-SVM method, but it predicted many false negatives with low sensitivity. HAMMER achieved high sensitivity but with little specificity. Considering *F* values and *MCC* values, the prediction results from BLAST and HAMMER are almost random.

**Table 2:** Classification performance.

Subtype	No. of cancer cases	No. of non-cancer cases	Method	Sn	Sp	Accuracy	F value	MCC
Western	37	211	Entropy-SVM	0.86	0.74	0.76	0.80	0.45
			BLAST	0.22	0.77	0.69	0.34	−0.01
			HMMER	0.94	0.005	0.14	0.009	−0.16
East Asian	47	240	Entropy-SVM	0.74	0.71	0.71	0.73	0.35
			BLAST	0.17	0.75	0.65	0.28	−0.07
			HMMER	1	0.003	0.19	0.05	0.06

The classification result and the contour plot (Figure 6) strongly support our hypothesis, i.e., the information of the selected residues in intervening regions can be used to classify the relation between CagA sequences and gastric cancer, although the difference between the profiles of cancer and non-cancer groups is not very strong.

### Comparison among Different Diseases

*H. pylori* infection is associated with most gastroduodenal diseases, among which gastric cancer is the most severe one causing more than 700,000 deaths worldwide every year [38]. Since *H. pylori* is a main risk factor of gastric cancer (GC), discovery of the mechanism of *H. pylori* mediating GC becomes a top priority task in this field. Comparing to other diseases, the diagnosis information of GC from public data is relatively accurate, and it is another important reason to focus on GC in this paper. Our studies are not limited to GC, though. We also tried to evaluate the relations between the variance of CagA sequences and different diseases.

Since most data were collected from public databases without accurate diagnosis information, before applying our method to CagA data, we manually curated the disease annotations for all strains by reviewing the literature. Table S1 lists the distributions of major diseases for both the Western and the East Asain subtype groups. Due to the limitation of strain numbers of some diseases, such as atrophic gastritis (AG) and gastric ulcer (GU), we eventually picked chronic gastritis (CG) and duodenal ulcer (DU) as the control groups for evaluation. The DU group in the East Asian subtype contains 79 strains, and a bootstrapping procedure was applied to all other groups to make the same number of strains as the East Asian DU group. This step guarantees all comparisons on the same scale, since the value of combinatorial entropy depends on the number of sequences. We used Formula (3) to calculate the entropy difference of each position between GC and CG/DU groups, and then added up all entropy differences as the total difference between GC and CG/DU groups, as shown in Table S2. By comparing results between two groups within the same geographic subtype (East Asian or Western subtype), it is consistent with the clinical view that gastritis has stronger relations to cancer than to DU [39] (generally, gastritis cases might contain some unreported or undiagnosed chronic atrophic gastritis and intestinal metaplasia cases, with which patients have a high risk to develop GC). By considering the same disease-pair between two geographic subtypes, it also explained the virulent difference between the East Asian and the Western subtypes. In addition, due to the high similarity between different disease groups of the East Asian subtype, even with more data, we still cannot reach the same classification accuracy as the Western subtype group.

Based on the above results, CagA sequences show potential to distinguish multiple gastroduodenal diseases. In order to evaluate the classification performance, we used DU group to replace non-Cancer group, and then applied the whole classification procedure again without bootstrapping, since those two diseases groups have comparable sizes. Table S3 shows the classification results. Although from the clinical point of view, DU has the negtive correlation with GC among all gastroduodenal diseases [40], the classification performance of two subtype groups was only slightly improved. Thus cancer-related CagA strains might have some unique sequence patterns comparing to all other gastroduodenal diseases. Hence, tuning a subset of the control group may not be able to improve the classification accuracy.

## Discussion

Although research indicates that there are sequence markers to differentiate between cancer group and non-cancer group, the major profiles of those two groups are too similar to distinguish by using traditional methods since the CagA sequences are overall highly conserved. Therefore, we focused on identifying the informative residues, quantifying information of these selected residues, and then using it to design a classifier that can predict whether a new sequence belongs to the cancer group or the non-cancer group. This method not only sheds light on the relations between CagA sequences and gastric cancer, but also may provide a useful tool for gastric cancer diagnosis or prognosis.

The mechanisms of *H. pylori* causing the different gastroduodenal diseases are still unclear, however it is likely that various gastroduodenal diseases caused by *H. pylori* infection share some sequence patterns in the intervening regions. Small variations of amino acids in those important residues might lead to the virulence variance of CagA strains resulting in different gastroduodenal diseases. While CagA could be a marker for detecting potential cancer risk, using CagA alone to distinguish all gastroduodenal diseases is not realistic. As a future study, we will develop new models that differentiate various gastroduodenal diseases from cagA and other genes.

## Supporting Information

Table S1 Number of strains in each disease.
(DOC)

Table S2 Total entropy difference between gastric cancer and two other diseases groups.
(DOC)

Table S3 Classification performance between gastric cancer and duodenal ulcer groups for both the Western and the East Asian subtypes.
(DOC)
