# Supplementary material for: Risk Assessment of Gastric Cancer Caused by Helicobacter pylori Using CagA Sequence Markers
Source: PLoS One. 2012 May 15;7(5):e36844. doi: 10.1371/journal.pone.0036844 (PMC3352932; doi:10.1371/journal.pone.0036844)
Supplement: Table S1 — Number of strains in each disease. (DOC) [file pone.0036844.s001.doc]

|  | GC | AG | CG | GU | DU | Other | Total |
| --- | --- | --- | --- | --- | --- | --- | --- |
| East Asian | 47 | 49 | 45 | 47 | 79 | 20 | 287 |
| Western | 37 | 8 | 44 | 14 | 50 | 95 | 248 |

GC: Gastric cancer; AG: Atrophic gastritis; CG: Chronic gastritis; GU: Gastric ulcer; DU: Duodenal ulcer
