# Supplementary material for: Risk Assessment of Gastric Cancer Caused by Helicobacter pylori Using CagA Sequence Markers
Source: PLoS One. 2012 May 15;7(5):e36844. doi: 10.1371/journal.pone.0036844 (PMC3352932; doi:10.1371/journal.pone.0036844)
Supplement: Table S2 — Total entropy difference between gastric cancer and two other diseases groups. (DOC) [file pone.0036844.s002.doc]

|  | GC vs CG | GC vs DU |
| --- | --- | --- |
| Western | -166.65 | -536.42 |
| East Asian | -57.18 | -244.03 |

GC: Gastric cancer; CG: Chronic gastritis; DU: Duodenal ulcer
