# Supplementary material for: Risk Assessment of Gastric Cancer Caused by Helicobacter pylori Using CagA Sequence Markers
Source: PLoS One. 2012 May 15;7(5):e36844. doi: 10.1371/journal.pone.0036844 (PMC3352932; doi:10.1371/journal.pone.0036844)
Supplement: Table S3 — Classification performance between gastric cancer and duodenal ulcer groups for both the Western and the East Asian subtypes. (DOC) [file pone.0036844.s003.doc]

| Subtype | No. of GC cases | No. of DU cases | Sn | Sp | Accuracy | F value | MCC |
| --- | --- | --- | --- | --- | --- | --- | --- |
| Western | 37 | 50 | 0.86 | 0.82 | 0.84 | 0.84 | 0.68 |
| East Asian | 47 | 79 | 0.68 | 0.73 | 0.71 | 0.71 | 0.41 |
